# Economics of Using GnRH on Day 5 After Timed Artificial Insemination in a Modified Double-Ovsynch Protocol at a Low-Fertility Dairy Farm

**DOI:** 10.3390/vetsci12070648

**Published:** 2025-07-08

**Authors:** Silviu-Ionuț Borș, Adina-Mirela Ariton, Alina Borș, Amalia-Ioana Hârbu, Vasile Vintilă

**Affiliations:** 1 Research and Development Station for Cattle Breeding Dancu, 707252 Iasi, Romania; amariton@yahoo.ro (A.-M.A.); vasilevintilais@gmail.com (V.V.); 2 “Ion Ionescu de la Brad” Iasi University of Life Sciences, 700490 Iasi, Romania; alina.bors@iuls.ro (A.B.); amalia.harbu@iuls.ro (A.-I.H.)

**Keywords:** anoestrus, dairy cows, Double-Ovsynch, accessory corpus luteum, profitability

## Abstract

This study evaluates the effectiveness and financial impact of treating anoestrus dairy cows using a modified DO protocol. Among the 350 cows included in the study, 142 (40.6%) were identified as anoestrus. The average daily milk production, days in milk at artificial insemination (AI), number of AIs, and parity were 33.8 ± 6.8 kg, 152.6 ± 24.4 days, 1.3 ± 0.2 AIs, and 1.9 ± 0.8 lactations, respectively. To assess embryo survival in anoestrus dairy cows undergoing the modified DO protocol, two groups were established. The experimental group (E group, *n* = 74) received the GnRH agonist gonadorelin five days after TAI as part of the DO protocol. The control group (C group, *n* = 68) received only the DO protocol. The pregnancy rates and cumulative pregnancy rates were significantly higher in the E group (35.1% and 56.8%) compared with the C group (26.5% and 45.6%, *p* < 0.05). A binary logistic regression analysis indicated that the interaction between treatment and anoestrus status considerably affected the pregnancy rate (*p* < 0.001) and the occurrence of accessory corpus luteum (aCL, *p* < 0.0001). The UW-DairyRepro$ decision support tool utilized in this study, indicated that implementing this approach could increase the net present value (NPV) by USD 54.2/anoestrus cow/year.

## Simple Summary

In the dairy cattle industry, the establishment and maintenance of pregnancy are critical factors influencing herd profitability. Researchers have concentrated their efforts on developing strategies aimed at enhancing fertilization rates and mitigating the adverse effects associated with poor embryo survival, which can ultimately lead to embryo resorption. Studies estimate that very early, early, and late embryonic losses in dairy cows can range from 40% to 60%, underscoring the severity of this issue. Embryo losses in anoestrus dairy cows represent a major challenge for reproductive success in the dairy industry. The inability of anoestrus cows to exhibit normal reproductive behaviors can severely limit conception rates and overall productivity within a herd. In light of this challenge, our study examines the effectiveness of a modified Double-Ovsynch (DO) protocol. This innovative approach incorporates the supplementary administration of a single dose of the GnRH agonist dephereline on day 5 post-timed artificial insemination (post-TAI) to induce corpus luteum (CL) formation and increase the progesterone level. The goal of this adjustment is to improve profitability by decreasing embryo losses, specifically in anoestrus dairy cows.

## 1. Introduction

Over time, low fertility in dairy cows has had a negative economic impact on the dairy industry. To achieve high profitability in the dairy industry, effective reproductive management is essential and hinges on four key factors. Firstly, timely AI should be carried out immediately after the voluntary waiting period. Secondly, increasing the pregnancy rate per AI (P/AI) is crucial. Thirdly, it is important to promptly re-inseminate cows that have not become pregnant. Lastly, efforts should be made to reduce pregnancy losses. Collectively, these metrics have a significant influence on the 21-day pregnancy rate, which is closely tied to the overall economic performance of dairy herds [1,2,3,4].

The most commonly used strategy to improve fertility is Ovsynch (GnRH1-7d-PGF2α-56 h-GnRH2-16 h-TAI), which combines GnRH and PGF2α to synchronize ovulation, thereby facilitating TAI [5]. Enhanced ovulatory responses to the first GnRH (GnRH1) of Ovsynch have been shown to positively influence P/AI in lactating dairy cows [6,7]. Building on this premise, many TAI programs have subsequently been developed. The main goal of these is to develop pre-synchronization protocols before initiating the Ovsynch protocol. Thus, anovular cows showed significant improvements when strategies initiating the CL development were implemented before starting the Ovsynch protocol [8].

Gonzalez et al. [9] proposed that implementing a targeted reproductive program could decrease the reliance on external hormones. In their study, cows that did not exhibit estrus alerts prior to the end of the voluntary waiting period were subjected to the DO protocol. Cows lacking estrus alerts may benefit from hormonal interventions; however, further research is necessary to validate this assumption. Results from a meta-analysis of 27 studies, involving 63 herds, confirmed that DO protocol resulted in a higher first-service P/AI in primiparous cows (51.4%) compared with prostaglandin-only treatment (43.4%). However, this difference was not observed in multiparous cows, where prostaglandin only showed a higher percentage (41.4%) compared with DO protocol (39.2%) [10].

Clinically healthy cows often experience embryo loss, followed by resorption, within the first 30 days of pregnancy. Progesterone deficiency plays a significant role in embryo loss, a major cause of low reproductive performance in dairy cows. Dairy cows exhibit increased metabolic activity, resulting in an accelerated rate of progesterone (P4) degradation, which leads to reduced blood concentrations [11,12]. Treatment with GnRH or hCG, administered between days 5 and 7 after AI, resulted in the formation of aCL in 70% to 90% of cows [13,14] and led to increased circulating P4 levels [14]. Several studies have shown that the induction of aCL is associated with increased P4 concentrations and improved conception rates in dairy cows [15,16,17,18,19].

In this context, it is important to investigate whether administering GnRH early in the luteal phase of a modified DO protocol enhances reproductive efficiency in cows with expected suboptimal fertility. Thus, our study aimed to identify the reproductive and economic impact of the modified DO protocol by adding GnRH on day 5 after TAI in anoestrus dairy cows.

## 2. Materials and Methods

### 2.1. Ethical Approval

All dairy cows were treated in accordance with the European Union directives on the protection of animals used for scientific purposes (Directive 2010/63/EU). The experimental protocol received approval from the Ethics Committee of the Faculty of Veterinary Medicine at the University of Life Sciences in Iasi, Romania. Efforts were made to minimize handling and stress for the animals.

### 2.2. Cattle Herd Management and Study Design

A Northeastern Romanian Holstein–Friesian dairy herd served as the subject of this study. The average number of lactating cows in the herd was 350 during the study period, with an average milk yield of 9500 kg/cow/305 days. The cows were housed in free-stall barns featuring concrete floors and straw bedding. They were fed a total mixed ration twice daily and had access to water at all times. Throughout the study, the farm milked approximately 350 cows twice a day, at 4:00 AM and 4:00 PM, resulting in a daily average milk production of 31.1 kg/cow/day.

The study involved 142 anoestrus Holstein cows, which were divided into two groups: the control group (C group, *n* = 68) and the experimental group (E group, *n* = 74). The cows were evenly distributed between pens based on days in milk (DIM) and parity. Information about calving dates, breeding dates, and DIM was obtained using AfiMilk management software v5.6.3 (AfiMilk, Kibbutz Afikim, Israel). Anoestrus cows were identified from the AfiMilk (AfiMilk, Kibbutz Afikim, Israel) weekly report. The anoestrus group included all cows that did not show any signs of estrus by day 60 after calving, as well as those that did not exhibit estrus by day 45 following AI. To prevent any recording errors, all cows diagnosed with other disorders were excluded from this study. Consequently, cows with conditions such as ovarian cysts or chronic endometritis were not included, totaling 142 animals. To monitor estrus, we obtained daily reports from AFIMILK management software v5.6.3 (AfiMilk, Kibbutz Afikim, Israel) and conducted daily veterinary visits. Each visit involved at least three hours of visual examination for signs of estrus in the herd, including one and a half hours in the morning and one and a half hours in the afternoon. Signs suggestive of estrus in a cow include behaviors such as attempting to mount other cows, chasing herd mates, showing restlessness, chin resting, sniffing the vaginas of other cows, bellowing, experiencing congestion, relaxing, and having a mucus discharge from the vulva. The definitive indicator of estrus is the observation of a cow standing while being mounted.

Both groups received a standard DO protocol, (GnRH-7d-PGF2α-3d-GnRH-7d-GnRH-7d-PGF2α-56h-GnRH-16h-TAI) with the difference that in the E group, a single dose of GnRH agonist (dephereline: gonadorelin acetate [6-DPhe]; Gonavet Veyx, Veyx-Pharma GmbH, Schwarzenborn, Germany) was administered in day 5 after TAI.

An experienced veterinarian conducted all ultrasound examinations and hormone injections. To evaluate ovarian structures and diagnose pregnancy, ultrasound scanning of the uterus and ovaries was performed using a 5–7.5 MHz rectal convex probe (Iscan2 ultrasound scanner, Draminski, Szabruk, Poland). Pregnancy was confirmed based on visualization of an anechoic fluid-filled uterine horn and the presence of an embryo, along with a CL in the corresponding uterine horn. When the number of corpora lutea exceeds the number of embryos, the presence of an additional CL or multiple corpora lutea supports the pregnancy [20]. We also recorded the number of corpora lutea at the time of pregnancy diagnosis, which was approximately 45 days after AI. Treatment with GnRH or hCG after AI enhances the production of aCL [21,22]. Consequently, any additional CL observed is referred to as aCL. Pregnancy was confirmed on day 90 after AI. All pregnant cows that exhibited signs of estrus between 45 and 90 days after AI and were found to have no detectable fetus during ultrasound evaluation were diagnosed with pregnancy loss. Additionally, on day 90, all cows without a confirmed pregnancy were also diagnosed with pregnancy loss. The conception rate represents the proportion of cows diagnosed as pregnant following the TAI service. The cumulative conception rate accounts for the proportion of cows diagnosed as pregnant, considering additional inseminations that occurred after the TAI service, but only if they took place within 21 days of the initial TAI service.

### 2.3. Economic Analysis

A total of 142 anoestrus dairy cows from a herd of 350, producing an average of 9500 kg of milk per cow over 305 days, were simulated using the UW-DairyRepro$ decision support tool [23]. The simulation included modifications as described by Giordano et al. [24] to assess the economic impact of treating cows with the GnRH agonist gonadorelin, administered 5 days after TAI in a modified DO protocol. The reproductive program simulated for the anoestrus cows was similar in both groups (TAI-DO protocol; subsequent AI—heat breeding), with the difference of using one supplementary dose of GnRH agonist 5 days after TAI in the E group. The following herd, economic, and reproductive parameters were included: average body weight (1763 lb), involuntary culling (15.5%), mortality rate (4%), stillbirth (7.7%), milk price (USD 28.6/cwt), cost feed lactation (USD 0.08/lb DM), the dry period fixed cost (USD 0.06/lb DM), female calf value (USD 200), male calf value (USD 100), the heifer replacement value (USD 2000), residual value (USD 0.526/lb), the voluntary waiting period (60 days in both groups), estrus cycle duration (22 days in both groups), maximum day in milk for breeding (300 days), do-not-breed minimum milk 50 lb/day, pregnancy loss (6.5% for C group and 7.1% for E group), days in gestation first pregnancy check (45 days), days in gestation second pregnancy check (90 days), conception rate TAI service (26.5% for C group and 35.1% for E group), cumulative conception rate (45.6% C group and 56.8% E group). The costs of reproductive programs included insemination cost (semen USD 16/cow, labor USD 5/cow), ultrasound pregnancy check (USD 100/h), synchronization—labor for injection at USD15/h, hormones (GnRH USD 2.6/dose, PGF USD 2.6/dose). The costs of a DO protocol were taken into account for both groups in the simulation program. The model estimated net present value (NPV; USD/cow/year) differences for the reproductive programs consisting of improved pregnancy rates following treatment with GnRH agonist gonadorelin 5 days after TAI in anoestrus dairy cows.

### 2.4. Statistical Analysis

Binary logistic regression was conducted, specifically, the logistic procedure in PASW Statistics for Windows Version 21 (SPSS Inc., Chicago, IL, USA), to investigate the effects of treatment on various dependent variables, such as conception rates, cumulative conception rates, aCL, and pregnancy loss. The analysis was adjusted for several factors, including lactation status, days in milk, milk yield, type of estrus, anoestrus, sire, and technician. Odds ratios and 95% confidence intervals were calculated using estimates and Wald 95% limits. To ensure that only significant factors were included in the model, we applied a backward elimination method to evaluate the explanatory variables and their interactions [25]. A significance level of *p* < 0.05 was established. The results are presented as means ± standard deviation (SD). The dependent variables analyzed included aCL, pregnancy loss, and the number of cows that were exclusively pregnant.

## 3. Results

Between August 2024 and April 2025, 142 anoestrus cows from a dairy herd were divided into two groups based on factors such as parity, AI services, milk production, and body condition score, as these elements can affect conception rates. According to Table 1, there were no significant differences (*p* > 0.05) in these independent factors between groups C and E. Daily milk yield, days in milk at AI, the number of services, and lactation were 33.8 ± 6.8 kg (from 30.6 to 36.4 kg), 152.6 ± 24.4 days (from 118 to 171 days), 1.3 ± 0.2 AIs (from 1 to 3 AIs), and 1.9 ± 0.8 lactations (from 1 to 4 lactations), respectively (mean ± SD, ranges between parentheses). In total, 142 cows (40.6%) of 350 cows with 3.3 ± 0.1 BCS and 124.6 ± 24.4 DIM were recorded as anoestrus cows. The conception rate, aCL, cumulative conception rate, and pregnancy loss in the studied cows were, respectively, 31% (44/142), 44.4% (63/142), 51.4% (73/142), and 6.8% (5/73) (Table 1). In the analysis of the dependent variable, we observed significant differences between the two groups (C group and E group) regarding several outcomes. The conception rate was lower (26.5%) in the C group compared with the E group (35.1%, *p* < 0.05). Additionally, there was a notable difference in the formation of aCLs, with rates of 10.3% in the C group versus 75.7% in the E group (*p* < 0.05). Furthermore, the cumulative conception rate was also lower in the C group (45.6%) compared with the E group (56.8%, *p* < 0.05).

The interaction between the therapy and embryo loss significantly affected the conception rate, as indicated by the odds ratio analysis (Table 2). This suggests that anoestrus cows in the E group were 1.5 times more likely to remain pregnant compared with the control group. Additionally, the odds ratio revealed that treated cows were 7.35 times more likely to have aCL compared with those in the control group (Table 3).

The simulation program used in this study indicated that the net present value (NPV/cow/year) for the E group was USD 54.2 higher than that for the C group (Table 4), due to the improvement in the income over feed cows, reproductive program cost and calf value. This investigation also assessed the improvement in conception rates achieved by treating cows with the GnRH agonist gonadorelin five days after TAI service. Implementing this strategy to enhance conception rates in anoestrus cows at a dairy farm with 350 cows can result in a profit of USD 7696/herd/year (EUR 6476/herd/year).

## 4. Discussion

One effective strategy reported for inducing cyclicity in anoestrus dairy cows involves the implementation of an Ovsynch protocol before a further consecutive Ovsynch protocol, known as DO protocol. This approach serves to presynchronize and stimulate cyclicity in the cows before they are subjected to the standard breeding Ovsynch protocol. Research conducted by Souza et al. [26] and expanded upon by Wiltbank et al. [27] highlights the advantages of this protocol. The findings underscore the potential of the DO protocol as a more effective strategy for improving reproductive performance in dairy herds experiencing issues with anoestrus cows.

The use of presynchronization with the Ovsynch protocol before initiating a modified version of the Ovsynch, which includes administering GnRH five days after TAI, may initially appear excessive. However, our primary goal is to develop effective protocols that significantly enhance fertility rates in lactating dairy cows, especially in anoestrus ones. This can be achieved by carefully optimizing follicle size and creating favorable hormonal conditions. In addition to these measures, we are also focusing on improving the progesterone environment by inducing aCL formation within the reproductive cycle to minimize the risk of pregnancy losses. During this study, we aimed to rigorously evaluate the hypothesis that modifying the established DO protocol by incorporating a GnRH injection on the fifth day after TAI would result in higher conception rates and improved economic outcomes. Our comparison specifically looked at the effectiveness of this modified approach against the traditional DO protocol in cows with known low reproductive performance. By investigating this modification, we hope to identify strategies that not only increase fertility but also offer better economic viability for dairy producers.

Our primary hypothesis of this research was that the conception rate in anoestrus cows can be significantly enhanced by employing a modified DO protocol, particularly through the administration of GnRH on day 5 following TAI. This approach aims to address the challenges associated with low fertility rates in anoestrus cows, ultimately leading to improved reproductive outcomes in dairy cows. By strategically timing the GnRH injection, we anticipate a more synchronized hormonal response that could facilitate ovarian function and enhance the likelihood of a successful conception.

In the modified DO treatment group, the conception rate was significantly higher compared with the control group, with rates of 35.1% versus 26.5%. Additionally, the cumulative conception rate also showed a marked increase, standing at 56.8% for the E group compared to 45.6% for the control. These results align closely with findings from Herlihy et al. [8], who studied cyclic cows and reported a conception rate of 34.7% for those undergoing the DO protocol, in contrast to a rate of 36.5% for the Presynch–Ovsynch protocol. This demonstrates that the optimized management strategies employed in the modified DO group may indeed enhance reproductive outcomes in anoestrus cows.

In our study, we observed that both the conception rate and cumulative conception rates in DO anoestrus cows from C group were significantly lower than the rates reported by Herlihy et al. [8]. Specifically, while Herlihy et al. [8] reported a conception rate of 34.7%, our findings indicated a rate of 26.5%, highlighting a noteworthy difference. This suggests that the DO protocol may have limitations in enhancing fertility outcomes in anoestrus cows compared with previously established methods. Differences in the criteria used to define anoestrus cows could account for the observed variations in the data. Factors such as specific progesterone thresholds, duration of inactivity, and the age or reproductive history of the cows may influence how anoestrus is identified and interpreted across different studies or farms. Additionally, variations in environmental conditions and management practices could further impact the definition and detection of anoestrus, leading to discrepancies in reported results. Further investigation is necessary to understand the underlying factors contributing to this reduced performance and to optimize TAI protocols for anoestrus cows.

Previous studies have consistently shown that utilizing a DO protocol can substantially improve the first postpartum pregnancy rate, especially in cyclic heifers and primiparous cows [4,28,29], but few of those studies have evaluated the reproductive efficiency of low-fertility dairy cows submitted to Presynch–Ovsynch or a Double-Ovsynch scheme for first service [30]. This enhancement in reproductive performance is particularly notable in comparison to other synchronization protocols, which may not yield similar results. For instance, in trials examining various synchronization strategies, double synchronization has been shown to optimize the timing of AI, thereby increasing the likelihood of conception during the early postpartum period. However, it is important to note that among multiparous cows, no significant difference in pregnancy rates was observed between the double synchronization method and other established protocols. This suggests that while the double synchronization strategy may be advantageous for heifers and primiparous cows, its benefits may not extend to all categories of cows within the herd [8].

The second hypothesis of this research posits that the conception rate can be significantly enhanced through the induction of aCL formation via the administration of GnRH on day 5 following TAI in a modified DO protocol. This intervention is expected to facilitate the maintenance of pregnancy by promoting luteal activity, thereby potentially reducing pregnancy losses experienced during the critical first 45 days post-TAI. By optimizing luteal function, the study aims to establish a more favorable reproductive environment, ultimately increasing overall fertility rates in dairy cows. In our study, we found that administering GnRH agonist on day 5 following the TAI protocol significantly increased the proportion of aCL to 75.7% in the E group, compared with only 10.3% in the C group. This finding suggests that the timing of GnRH administration plays a critical role in enhancing luteal activity, potentially influencing fertility outcomes in our subjects. Further research could explore the underlying mechanisms contributing to this increase and its implications for reproductive management practices. Doležel et al. [31] found that administering gonadorelin to cows on day 5 post-AI significantly enhanced the formation of the aCL. This timing was shown to be more effective than administering gonadorelin on days 6 or 7 after AI. The results indicate that early intervention with gonadorelin can optimize reproductive outcomes in cattle by promoting the development of the aCL, which is crucial for maintaining pregnancy. Yan et al. [32] reported that progesterone treatment between 3 and 7 days after AI was beneficial, while earlier or later treatments showed no advantages. Additionally, Besbaci et al. [33] demonstrated that administering GnRH 10 days after AI could also result in pregnancy. In our previous study [34], we observed that administering GnRH 7 to 14 days after AI improved reproductive activity in repeat breeder cows. However, in a recent study [35] conducted with high-yielding cows averaging 46 kg of milk per day, we found that the conception rate in cows subjected to the modified Ovsynch protocol—receiving GnRH on day 5 after TAI—was similar to that of cows undergoing the standard Ovsynch protocol. Probably, the difference in milk output between the two study farms (46 vs. 31 kg of milk per day) influenced the outcome of GnRH treatment on day 5 after TAI. This change in milk production could have a significant impact on the differences between the current and earlier study [34]. Furthermore, comparing the current study with other investigations [34,36], we discovered that inducing aCL with GnRH administration significantly improved the reproductive performance of low-fertility dairy cows. These findings may have important implications for improving fertility management practices in the dairy industry.

Pregnancy losses represent a critical challenge in dairy production systems, leading to substantial economic repercussions [37]. Thus, this research presents a third hypothesis: in low fertility dairy farms, overall economic performance can be enhanced by implementing a modified DO protocol combined with the administration of GnRH agonist on day 5 following TAI. This approach aims to improve reproductive efficiency by optimizing the synchronization of ovulation and enhancing the likelihood of successful conception in cows that are experiencing low fertility rates. By refining the timing and methods of hormone administration, we anticipate a positive impact on both productivity and profitability in the dairy cattle industry. Research conducted by Cabrera in 2012 and 2013 [1,38], along with further studies by Cabrera and Fricke in 2021 [39], highlight that these losses not only affect the immediate milk production capabilities, but also have long-term implications on herd fertility, management costs, and overall farm profitability. In the current study, we analyzed the contribution to net value by breaking down income into various components: feed costs, replacement costs, reproductive program costs, and the value of calves. The advantage observed in the modified E group is likely to be attributable to the increased income in relation to feed costs, reproductive costs (specifically the administration of GnRH on day 5 after TAI within the modified DO protocol), and the value of the calves. This strategy may also enhance other reproductive management options, as it has been found to yield a positive net present value for the farm. Our research supports the hypothesis that administering GnRH agonists on day 5 after TAI in the modified DO protocol can improve fertility in anoestrus cows and enhance farm profitability. The GnRH agonist treatment increased the likelihood that pregnant cows would develop an aCL, which could enhance fertility in anoestrus cows undergoing TAI protocol. This suggests that by improving the conception rates of 142 anoestrus cows on a farm with 350 dairy cows, there could be an annual profit of USD 7696 per herd (approximately EUR 6476/herd/year). These results are superior compared to previous reports, in which a profit of USD 5677.6/heard/year was obtained by inducing aCL formation in repeat breeder cows [34]. A meta-analysis study carried out by Chen et al. [36] comparing controls, GnRH, and hCG administration for aCL formation provided solid evidence that embryo survival in the first month after estrus is essential to improving pregnancy rates and economic benefits. The reproductive performance of recipient cows with low fertility was significantly improved by inducing aCL using GnRH or hCG. Thus, under certain circumstances, treatment with a GnRH agonist early in the luteal phase after AI could have positive benefits on reproductive performance and economic returns of dairy farms. Addressing the underlying causes of pregnancy loss is essential for improving reproductive efficiency and reducing financial losses within the dairy industry.

## 5. Conclusions

Anoestrus in dairy cows poses a significant challenge to profitability within the industry, leading to decreased reproductive efficiency and increased economic losses. In our study, we implemented the DO protocol, which has demonstrated remarkable efficacy in enhancing reproductive performance in dairy herds. By strategically introducing GnRH on day 5 post-TAI within the DO protocol, we observed a notable increase in conception rates, averaging around 9%. This modification not only boosted conception rates, but also give a substantial increase in farm profitability, estimated at approximately USD 7696 per herd annually (approximately EUR 6476/herd/year). Our findings indicate that this approach effectively increased the number of corpora lutea in anoestrus cows undergoing TAI protocols, which, in turn, reduced embryo losses during critical developmental stages. As a result, this enhanced reproductive strategy can significantly improve overall reproductive programs, particularly in low-fertility dairy farms. By optimizing these reproductive protocols, producers can achieve better herd management and economic sustainability.

## Figures and Tables

**Table 1:** Independent variables for each group and the effects on different classes on each dependent variable (*n* = 142).

Treatment Groups	C Group (*n* = 68)	E Group (*n* = 74)	Total (*n* = 142)
Independent variable			
Parity	1.9 (±0.8)	1.8 (±0.9)	1.9 (±0.8)
DIM	146 (±23.8)	158.3 (±26.7)	152.6 (±24.4)
Milk yield (kg)	34.8 (±6.6)	32.7 (±5.4)	33.8 (±6.8)
Number of services	1.1 (±0.4)	1.4 (±0.3)	1.3 (±0.2)
BCS	3.3 (±0.1)	3.4 (±0.1)	3.3 (±0.1)
Dependent variable			
No. pregnant	18	26	44
No. open	50	48	98
Conception rate (%)	26.5 ^b^	35.1 ^a^	31
Accessory CL (%)	10.3 ^b^	75.7 ^a^	44.4
Cumulative conception rate (%)	45.6 ^b^	56.8 ^a^	51.4
Pregnancy loss (%)	6.5	7.1	6.8
Culling rate (%)	14.7	16.2	15.5

^a,b^ Group with different superscripts are significantly different at *p* < 0.05.

**Table 2:** Odds ratios of the conception rate variables included in the final logistic regression model (*n* = 142).

Factor	Class	*n*	% Pregnancy	Odds Ratio	95% Confidence Interval	*p*
Treatment	C group	18/68	26.5	Reference		
	E group	26/74	35.1	1.5	0.73–3.09	<0.001

R^2^ Nagelkerke = 0.14.

**Table 3:** Odds ratios of the aCL variables included in the final logistic regression model (*n* = 142).

Factor	Class	*n*	% aCL	Odds Ratio	95% Confidence Interval	*p*
Treatment	C group	7/68	10.3	Reference		
	E group	56/74	75.7	7.35	3.13–17.23	<0.0001

R^2^ Nagelkerke = 0.13.

**Table 4:** Contribution to net value.

Items	C Group	E Group	Difference
Total net value (USD/cow/year)	4493.6	4547.8	54.2
Income over feed cost (USD/cow/year)	4862.1	4910.9	48.8
Replacement cost (USD/cow/year)	−152.4	−155.3	−2.9
Reproductive cost (USD/cow/year	−272	−265.9	6.1
Calf value (USD/cow/year)	55.9	58.1	2.2

## Data Availability

The data that support the findings of this study are available upon reasonable request.

## Abbreviations

The following abbreviations are used in this manuscript:

DO	Double-Ovsynch
TAI	Timed artificial insemination
AI	Artificial insemination
aCL	Accessory corpus luteum
DIM	Days in milk
BCS	Body condition score
